# DNA methylation under the major depression pathway predicts pediatric quality of life four-month post-pediatric mild traumatic brain injury

**DOI:** 10.1186/s13148-021-01128-z

**Published:** 2021-07-12

**Authors:** Kuaikuai Duan, Andrew R. Mayer, Nicholas A. Shaff, Jiayu Chen, Dongdong Lin, Vince D. Calhoun, Dawn M. Jensen, Jingyu Liu

**Affiliations:** 1 Department of Electrical and Computer Engineering, Georgia Institute of Technology, Atlanta, USA; 2 Tri-Institutional Center for Translational Research in Neuroimaging and Data Science (TReNDS), Georgia State University, Georgia Institute of Technology, Emory University, 55 Park Place NE, 18th Floor, Atlanta, GA 30303 USA; 3 The Mind Research Network, Lovelace Biomedical and Environmental Research Institute, Albuquerque, USA; 4 Department of Computer Science, Georgia State University, Atlanta, USA; 5 Department of Psychology, Georgia State University, Atlanta, USA; 6 The Neuroscience Institute, Georgia State University, Atlanta, USA

**Keywords:** Pediatric mild traumatic brain injury, Depression, DNA methylation, Post-concussion symptom burden, Pediatric quality of life

## Abstract

**Background:**

Major depression has been recognized as the most commonly diagnosed psychiatric complication of mild traumatic brain injury (mTBI). Moreover, major depression is associated with poor outcomes following mTBI; however, the underlying biological mechanisms of this are largely unknown. Recently, genomic and epigenetic factors have been increasingly implicated in the recovery following TBI.

**Results:**

This study leveraged DNA methylation within the major depression pathway, along with demographic and behavior measures (features used in the clinical model) to predict post-concussive symptom burden and quality of life four-month post-injury in a cohort of 110 pediatric mTBI patients and 87 age-matched healthy controls. The results demonstrated that including DNA methylation markers in the major depression pathway improved the prediction accuracy for quality of life but not persistent post-concussive symptom burden. Specifically, the prediction accuracy (i.e., the correlation between the predicted value and observed value) of quality of life was improved from 0.59 (*p* = 1.20 × 10^–3^) (clinical model) to 0.71 (*p* = 3.89 × 10^–5^); the identified cytosine-phosphate-guanine sites were mainly in the open sea regions and the mapped genes were related to TBI in several molecular studies. Moreover, depression symptoms were a strong predictor (with large weights) for both post-concussive symptom burden and pediatric quality of life.

**Conclusion:**

This study emphasized that both molecular and behavioral manifestations of depression symptoms played a prominent role in predicting the recovery process following pediatric mTBI, suggesting the urgent need to further study TBI-caused depression symptoms for better recovery outcome.

**Supplementary Information:**

The online version contains supplementary material available at 10.1186/s13148-021-01128-z.

## Introduction

It is increasingly recognized that genomic factors may enhance prognostication for outcomes following traumatic brain injury (TBI) [1, 2]. Single-nucleotide polymorphisms (SNPs) associated with TBI outcomes [3–5] have underscored genes involved in neural repair and plasticity (e.g., brain-derived neurotrophic factor [BDNF]), cognitive reserve/behavior (e.g., solute carrier family 6 member 3), neurotransmitters (e.g., COMT/DRD2/ANKK1), and cytokines (IlIβ, IL1RN, IL6, IL1A TNFA). The results from research on polygenic effects are very promising [2, 6] but not conclusive. For instance, in a pediatric TBI cohort, a polygenic risk score derived from inflammatory genes (ACE, BDNF, IL-1RN, and NT5E) was associated with increased executive dysfunction and behavioral problems [2], while another study of military TBI cohort failed to show a relationship between polygenic risk scores for ten types of diseases and persistent post-concussion symptom (PPCS) [6].

In contrast, very few studies have examined the relationship between epigenetic changes and outcomes following TBI in clinical samples [7]. Most existing knowledge about epigenetic changes following TBI has been gained from animal studies. Epigenetic modification following TBI have been reported in various types of molecular mechanisms, including DNA methylation in animal models [8–10], microRNA in humans with TBI [11–13] and the upregulation of various transcription factors involved in the regulation of inflammatory and immune response, apoptosis, or the migration of neuronal progenitor cells in rats [14]. In particular, DNA methylation at BDNF promoters potentially played an important role in the anxiety-like behavior in rats following minimal TBI [9, 15].

Recently, Li conducted an epigenome-wide DNA methylation association study on the recovery outcome 1–6-day post-injury in a cohort of 120 severe TBI patients, but failed to identify any methylation sites passing the epigenome-wide significance level [16]. The main challenge with this type of analysis is the large number of tested CpG sites and weak effects carried by each site [2, 17, 18]. Thus, a more focused investigation of specific pathways may be an effective approach to investigate epigenetic involvement. For example, Nielsen and colleagues [19] have recently identified that methylation CpG sites in the apolipoprotein E (APOE) promoter region were related to the plasma APOE protein levels, and plasma APOE protein levels were associated with posttraumatic stress disorder symptom severity in a cohort of veterans with and without mild TBI (mTBI).

Besides genomic and epigenetic factors, demographic (e.g., age, sex) and clinical variables (e.g., degree of posttraumatic stress, psychological distress, pain) also play important roles in determining outcomes following mTBI [20, 21]. Specifically, both preexisting and posttraumatic affective disturbances have been shown to be one of the strongest predictors of outcome following mTBI [22]. Bombardier and colleagues [23] reported that 53% of mTBI participants (*N* = 297/559) met the diagnostic criteria for major depressive disorder (MDD) in at least one instance during a one-year follow-up period [1, 6, 8, 10, and 12 months], and mTBI participants with MDD had lower quality of life at one-year follow-up compared with those without MDD. Similar findings were also reported recently by Stein and Mac Donald [24, 25]. Moreover, a review paper on depression and depressive symptoms in pediatric TBI suggests that depression is likely a secondary outcome following pediatric TBI [26]. The underlying mechanism at the molecular level linking the clinical conditions (e.g., anxiety, depression, and stress) to worse outcomes following mTBI in humans has yet to be discovered. Understanding the biological basis of traumatically induced depression and the relationship with outcomes is critical given the increased risk for suicide rates for both concussed persons and athletes retrospectively diagnosed with chronic traumatic encephalopathy [27, 28].

This study was undertaken to investigated whether DNA methylation markers from the major depression pathway could predict post-concussive symptom burden and quality of life four-month post-injury in a pediatric mTBI (pmTBI) cohort beyond traditional demographic and clinical variables [21]. Methylation CpG sites in genes BDNF and APOE4 were also included as prior features in analyses given previous associations observed in the preclinical and clinical literature [9, 15, 29–35].

## Materials and methods

### Participants

Participants were from an ongoing study examining biomarkers in pmTBI [36]. Inclusion criteria for mTBI participants were based on the American Congress of Rehabilitation Medicine and the Zurich Concussion in Sport Group guidelines (see details in Supplemental Materials). Exclusion criteria included the history of previous head injury with greater than 30-min loss of consciousness, neurological diagnosis, most psychiatric disorders, autism spectrum disorder, intellectual disability, history of substance abuse/dependence or current use, contraindications for MRI, or non-English speaking. Healthy controls (HCs) were also free of diagnosis of attention-deficit hyperactivity disorder, a learning disability, or a recent mTBI (within six months). The study was approved by the University of New Mexico School of Medicine Institutional Review Board.

Based on the quality of acquired DNA material, completion of clinical measurements, and data quality control (details later on), the final sample included 110 pmTBI patients at the subacute (SA) visit (8–18 years; 14.90 ± 2.07 years old; 53 females; 7.26 ± 2.28-day post-injury), with 91 returning for the early chronic (EC) visit (14.80 ± 2.10 years old; 46 females; 130.08 ± 13.20-day post-injury; 122.45 ± 14.29 days between visits). In addition, 87 age-matched HC completed both SA and EC visits (14.93 ± 2.00 years old; 36 females; 124.84 ± 15.67 days between visits). The 197 participants were from 171 one-member families and 13 two-member families (exclusively of HC). One hundred and sixty-four participants reported themselves as White, and 5 as Black or African-American (detailed race information can be found in Supplemental Materials).

### Clinical and cognitive measures

Clinical assessments were administered during SA visit (1–10-day post-injury) and EC visit (approximately four-month post-injury), using a common data elements focused battery of clinical and neuropsychological measures. Retrospective ratings (i.e., estimation for one month ago) of symptoms and quality of life were also acquired at the SA visit (See Table S1 for retrospective clinical ratings across groups). Two primary clinical measures were included, the Post-Concussion Symptom Inventory (PCSI) and the Pediatric Quality of Life Inventory Generic Core (PedsQL). PCSI had self- and parental reports with lower PCSI values indicating fewer or less severe symptoms. The PedsQL is a self-report measure with larger values indicating better quality of life. It is a reliable outcome measure and can discriminate among children with TBI [37]. Secondary clinical measures consisted of the Patient Reported Outcomes Measurement Information System (PROMIS; anxiety, depression, and sleep scales), a self-report pain rating (0–10 Likert scale) and the Headache Impact Test (HIT-6). Additional injury characteristics, such as loss of consciousness (LOC), posttraumatic (PTA) and retrograde (RTA) amnesia, and the previous number of concussions, were also collected (see Supplemental Materials). Finally, substance use history for tobacco, alcohol, and cannabis were summarized based on Alcohol, Smoking, and Substance Involvement Screening Test (ASSIST). Days post-injury at SA visit were not significantly related to any of the above-mentioned clinical assessments at SA after false discovery rate (FDR) at *p* < 0.05 correction.

Cognitive estimates of premorbid abilities were obtained along with neuropsychological tests selected from specific subtests of the Delis–Kaplan Executive Function System (DKEFS) and age-appropriate versions of Wechsler scales. Raw scores were age-corrected and aggregated into composites for primary domains of attention and processing speed, along with secondary domains of working memory and executive function. Measures of processing speed, attention, visual learning, and working memory were acquired from the Cogstate battery (detection, identification, one card learning, and one back test, respectively) for both reaction time and accuracy.

### Classification of persistent post-concussion symptoms

Classification of PPCS from “recovered” patients at the follow-up visit four-month post-injury (EC) was determined based on self-reported PCSI data [38]. Briefly, individual HC and pmTBI PCSI data at the EC visit were summed, base 10 log-transformed to correct for non-normality, and further normalized to a standard normal distribution based on the HC EC data. A z threshold of 1.64 (the same threshold as in a previous study [38]) was used to classify patients as having PPCS, while those below the threshold as “recovered” patients. A clinical risk score for PPCS [21] was also calculated for all pmTBI patients using demographic and clinical data collected at the SA phase (see Supplemental Materials).

### DNA methylation data processing

Saliva samples were collected during the SA visit. DNA samples (see Supplemental Methods for details) isolated from saliva were used to generate methylation data by the Infinium Methylation EPIC array, covering over 850,000 CpG sites. A series of quality control steps were performed. Specifically, we removed individuals (1) with mismatched sex information between self-report and methylation data indicated, (2) with more than 1% of methylation sites missing (CpG sites with detection p-value larger than 0.05 were set as missing), and (3) three standard deviations away from the median based on the top four principal components (PCs) of the methylation (beta value) matrix. Using R package minfi [39], both methylated and unmethylated signals of the remaining participants were normalized based on the quantile-based normalization method. For methylation CpG sites, we excluded (1) CpG sites with more than 1% missing values; (2) CpG sites on sex chromosomes; (3) CpG sites coinciding with SNPs or at the single-nucleotide extension; (4) CpG sites potentially affected by cross-hybridization as provided in [26]. Totally, 754,160 methylation sites were retained after preprocessing. Missing beta values were imputed with the average of CpG sites. Batch (chip) effects were corrected by using the R package Combat [40]. Cell type (B cells, CD8 + T cells, monocytes, granulocytes, and buccal cells) proportions in saliva samples were estimated by using a previously proposed algorithm [41] with the reference methylation data collected from buccal epithelial cells (GSE46573) and other leukocyte cell types available in the minfi package [39]. The estimated proportions for B cells, CD8 + T cells, and monocytes were very close to 0. Proportions of granulocytes and buccal cells summed to one approximately. So, we used buccal cell proportion as a covariate to control for potential confounding effects from cell type proportion in this study.

A methylome-wide association study (MWAS) was performed on the batch-corrected beta values of 754,160 CpG sites to investigate pmTBI versus control difference using a mixed effect regression model:

a CpG site = diagnosis (pmTBI/control) + age + sex + BMI + race + buccal cell proportion + three principal components of methylation + family,

where family was modeled as a random effect and other predictors were modeled as fixed effects. Race (White, African-American or other) was coded as dummy variables. BMI represented body mass index. Principal components (PCs) decomposed from genomic methylation data have been used to control for potential confounding factors in genomic association analyses [42]. The change rate of eigenvalues suggested using the first five PCs, while PC 1 was highly collinear with buccal cell proportions (correlation *r* = 0.87) and PC 5 was associated with gender (*r* = 0.45, *p* = 4.18 × 10^–11^), race (*r* = − 0.33, *p* = 3.17 × 10^–6^), and PCSI at SA (*r* = 0.19, *p* = 0.02). Given that buccal cell proportions, gender, and race were already included in the model and PCSI at SA significantly related to the variables we are interested in (PCSI at EC with *r* = 0.44, *p* = 9.08 × 10^–10^ and PedsQL at EC with *r* = − 0.47, *p* = 3.94 × 10^–11^). To avoid PC 5 canceling out the partial individual variance we are interested in, we only controlled three principal components (PC 2–4) (the same for later models). Previous studies [43–46] have suggested using a standard deviation (SD) > 0.05 as a reasonable threshold for selecting reliable and variable CpG sites. We chose SD threshold of 0.1 for the selection of reliable and variable CpG sites because our estimated sample variance is likely larger than population variance due to the relatively small sample size of this study. This more conservative selection yielded 17,857 CpG sites. Derived methylation features included global methylation level and independent factors from the major depression pathway, as well as individual CpG sites in BDNF and APOE4 genes. The average beta values of all included epigenomic CpG sites were treated as a proxy of global methylation for each participant. The pmTBI versus control difference of the global methylation was investigated using the above-mentioned mixed effect model, and the only difference is that global methylation was treated as the dependent variable.

Given the primary focus on the predictive ability of the major depression pathway, we selected 278 genes annotated to affect major depression in the ingenuity pathway analysis database (IPA, http://www.ingenuity.com). Adding 20 k base-pair flanking regions for each gene, we mapped 223 CpG sites after quality control in our data. Lists of the selected 278 genes and mapped 223 CpG sites can be found in the Supplemental spreadsheet. A multivariate blind source separation method, independent component analysis [ICA [47]], was applied onto the 223 CpG sites of 110 pmTBI patients at the SA visit to capture maximally independent patient-specific methylation patterns. Methylation data (hereafter referred to as $\mathbf{X})$ were decomposed into a component matrix $\mathbf{S}$ and a loading matrix $\mathbf{A}$ ($\mathbf{X}=\mathbf{A}\times \mathbf{S}$). Each row of $\mathbf{S}$ is one independent component, and individual CpG sites contribute to the component differently. Each column of $\mathbf{A}$ is the loading vector, which represents the expression levels of the corresponding component across participants. The component number is estimated to be 30 based on Chen’s consistency method [48] and retained 96.95% of the data variance. Global methylation level and loading vectors in $\mathbf{A}$ were utilized as methylation features in the prediction model.

Since we were also interested in investigating CpG sites in BDNF and APOE4 genes for their ability to predict symptoms and outcomes following pmTBI, 13 methylation CpG sites under BDNF and APOE4 genes with standard deviations larger than 0.05 (see Table S2 for detail, they were not included in the selected 223 CpG sites because their standard deviations were less than 0.1) were included as prior methylation features [44, 49]. Given that only 13 CpG sites were covered by BDNF and APOE4 genes, we believe that support vector regression (SVR) with the least absolute shrinkage and selection operator (LASSO) model (explained in detail later) is able to capture effects of all linear combinations of 13 CpG sites. Thus, it is not necessary to apply ICA factorization to 13 CpG sites prior to SVR with LASSO.

### Outcome/symptom prediction models

**Fig. 1:**
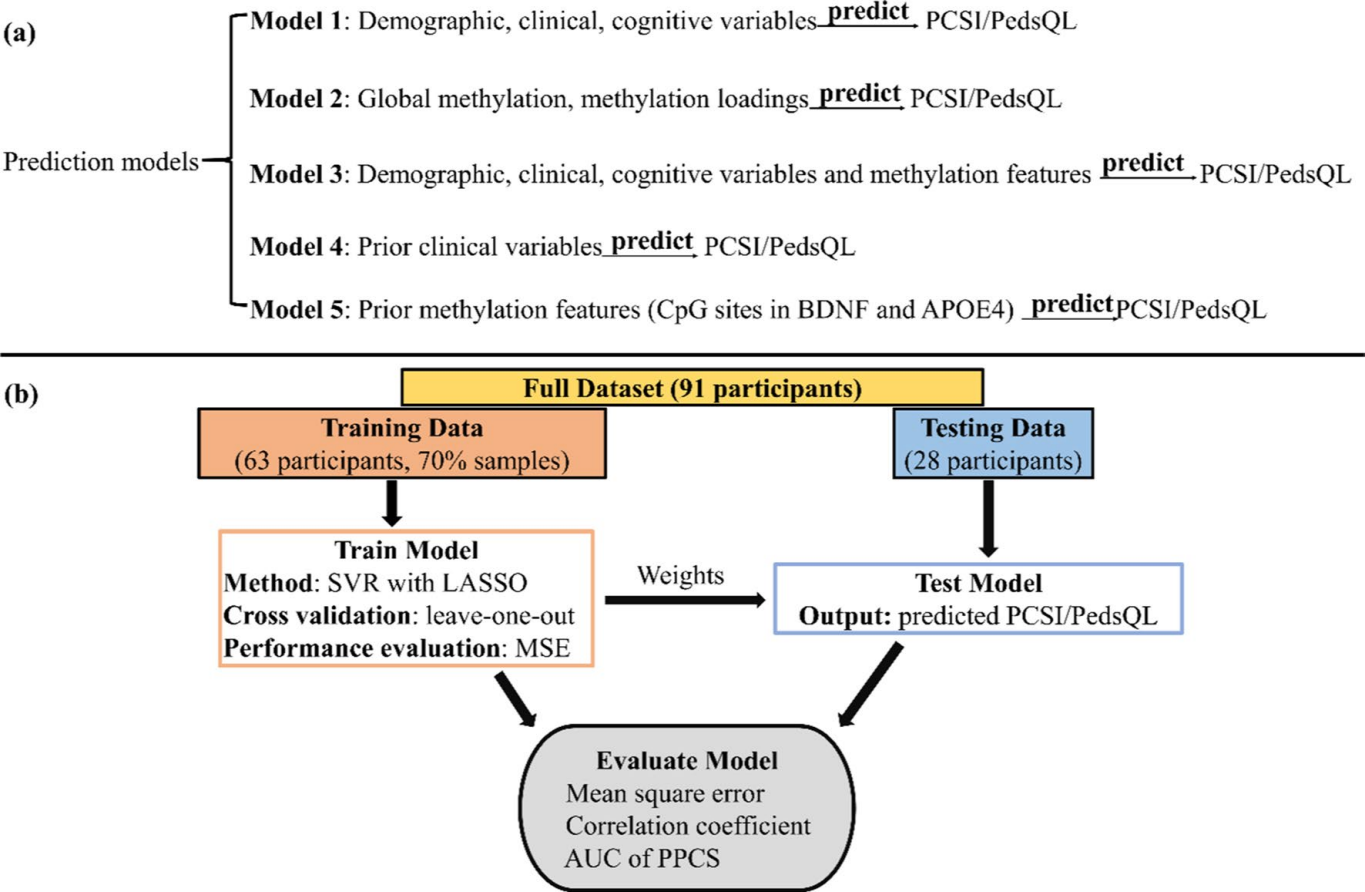
**a** Prediction models and **b** Diagram for predicting PCSI/PedsQL

In this study, we employed SVR with LASSO regularization to predict PCSI and PedsQL scores at four-month post-injury using data available at the SA visit. SVR with LASSO can select features of importance to achieve a stable regression outcome. We tested five different models (see Fig. 1a). Model 1 focused on all available features from demographic, clinical, and cognitive variables. Model 2 focused on DNA methylation features (i.e., global methylation level and 30 independent methylation components decomposed from the major depression pathway). Model 3 combined variables in Models 1 and 2, whereas Model 4 focused on behavioral variables subjectively selected to reflect the known key features of TBI. Model 5 focused on methylation CpG sites in BDNF and APOE4 genes. Thus, we named Models 1–5 as clinical, methylation, clinical + methylation, prior clinical, and prior methylation models, respectively. Each model was cross-validated with the training samples, and its performance was evaluated with a hold-out testing set (Fig. 1b). Among 91 pmTBI patients with information at both visits, 63 (around 70% samples, female/male = 32/31, age: 14.75 ± 2.12 years old) were utilized for training, with the remaining 28 participants (around 30% samples, female/male = 14/14, age: 14.93 ± 2.11 years old) used as a hold-out testing set. Mean square error (MSE) and correlation coefficient ($r$) between the predicted PCSI/PedsQL and true PCSI/PedsQL scores, receiver operating characteristic (ROC) curve for classifying PPCS versus recovered patients using the predicted PCSI/PedsQL score with all possible thresholds, and the area under the ROC curve (AUC) was estimated to reflect the accuracy of the prediction models.

The full demographic variables included age, sex, BMI, and duration between two visits. The full clinical variables included the previous number of concussions, LOC, PTA, RTA, depression, sleep disturbances, pain rating, and PCSI score at the SA visit. Cognitive variables included measures of processing speed, attention, visual learning, and working memory from the Cogstate battery, DKEFS battery, and Wechsler tests. In addition, history of substance use of tobacco, alcohol, and cannabis from ASSIST were also included. For the methylation model, global methylation level and loadings of 30 methylation components from the major depression pathway were used as predictors. For the prior clinical model, the features included the clinical risk score for PPCS [21], the previous number of concussions, LOC, PTA, and RTA, and days post-injury at the SA visit. For the prior methylation model, 13 CpG sites in BDNF and APOE4 genes were included as predictors. Each variable/predictor was scaled to be between 0 and 1 prior to SVR for all models.

Training a model includes three steps: tuning the regularizer λ for the LASSO, feature selection, and model fitting. We adopted the leave-one-out cross-evaluation to tune the parameter λ for LASSO regularization due to the small sample size. The λ producing the smallest MSE in the leave-one-out cross-evaluation was selected. With the selected λ, variables were included as final predictors for PCSI/PedsQL predictions when their weights from all cross-validation trials were significantly different from zero in Models 1 and 2. Model 3 included variables selected from Models 1 and 2 for fair comparisons with the accuracies achieved from Model 1 and 2. Features were fixed for Models 4 and 5 (prior clinical and methylation model). Model fitting used all selected variables and all samples in the training set to finalize model parameters (weights). When testing a model, the finalized model was applied to the hold-out testing samples to predict PCSI/PedsQL, and the prediction performance was evaluated by comparing the predicted PCSI/PedsQL values with the true values.

#### Secondary analyses

After finalizing the models, we then performed univariate analyses for each selected variable to better understand their characteristics, including associations with PCSI/PedsQL score at the EC visit, as well as differences between pmTBI and HC, if applicable. FDR at *p* < 0.05 was applied to correct for multiple comparisons.

For the identified clinical/cognitive variables, the pmTBI versus HC difference was tested using linear mixed-effect regression model (a) (Table 1). For the identified methylation components, we projected them into HC data and computed the corresponding loadings of controls (see Supplemental Materials for details). The pmTBI versus HC difference of methylation loadings and CpG sites in BDNF or APOE4 was tested with linear mixed-effect regression model (b) (Table 1). The associations between each identified methylation feature (e.g., global methylation, loadings of methylation components or CpG sites in BDNF or APOE4) and PCSI/PedsQL/depression symptom score at the EC visit were investigated for pmTBI participants with the linear regression model (c) (Table 1).

**Table 1:** Linear mixed/fixed effect regression models used in the secondary analyses

Model	Function	Response	Predictors	
			Fixed effect	Random effect
Model (a)	Test pmTBI versus HC difference of clinical/cognitive variables	A clinical or cognitive variable	Diagnosis (pmTBI/HC), age, sex, BMI, race	Family ID
Model (b)	Test pmTBI versus HC difference of methylation features	Loadings of a methylation component or a CpG site in BDNF/APOE4	Diagnosis (pmTBI/HC), age, sex, BMI, race, buccal cell proportion, PC 2–4	Family ID
Model (c)	Test the association between methylation features and PCSI/PedsQL/depression symptom score at EC within pmTBI patients	Global methylation or loadings of a methylation component or a CpG site in BDNF/APOE4	PCSI/PedsQL/depression symptom score at EC, age, sex, BMI, race, buccal cell proportion, PC 2–4	N/A
Model (d)	Test the association between depression symptom at SA/EC and loadings of 30 methylation components within pmTBI patients	Depression symptom at SA/EC	Loadings of 30 methylation components, age, sex, BMI, race	N/A

BMI represents body mass index. N/A denotes not available (same for Tables 2 and 3)

To test how the IPA-selected molecular features (i.e., all 30 methylation components decomposed from the major depression pathway) represented the depression symptom at SA and EC visits within pmTBI patients, we used the linear regression model (d) (Table 1). The methylation-fitted depression score was computed based on the regression coefficients of 30 methylation components, and its correlation with the true depression symptom score was further evaluated.

For the identified methylation components, top CpG sites (with weights |*z*|> 2), their locations and z values, relations to the island, and annotated gene names were summarized. Correlations between saliva and brain methylation for top CpG sites were checked using the IMAGE-CpG tool (http://han-lab.org/methylation/default/imageCpG#) [50]. Moreover, the reliability of top CpG sites was examined by using the summary provided in [51], where the reliability of 438,593 CpG sites covered by the Illumina Human Methylation 450K chip was summarized.

Given that the whole methylome data were available, depression-related methylation feature selection was performed on 197 samples by applying a univariate MWAS on the depression symptom at SA. Specifically, each of the 17,857 CpG sites was regressed on the depression symptom at SA, with diagnosis (pmTBI/control), age, gender, BMI, race, buccal cell proportions and PC 2–4 as fixed effects and family ID as a random effect. Depression-related top CpG sites (*p* < 0.05) were then selected and used as the features for SVR with LASSO to predict PCSI and PedsQL scores at EC.

In addition, univariate MWASs on PCSI and PedsQL scores at EC visit were performed on the 63 training samples to select CpG sites potentially contributing to PCSI and PedsQL prediction, respectively. Specifically, each of the 17,857 CpG sites was regressed on PCSI/PedsQL score at EC, with age, gender, BMI, race, buccal cell proportions and PC 2–4 as fixed effects and family ID as a random effect. PCSI-related or PedsQL-related top CpG sites were then selected and used as the features for SVR with LASSO to predict PCSI or PedsQL scores at EC (see details in the Supplemental Materials).

## Results

Table 2 lists the demographic and primary outcome measures. Based on thresholded PCSI scores at EC visit, 23 pmTBI patients were classified as having PPCS, and the remaining 68 pmTBI as recovered.

**Table 2:** Demographic information, PCSI and PedsQL across groups at each eligibility phase

Variables	SA		EC	
	HC	pmTBI	HC	pmTBI
Number of participants	87	110	87	91
Sex (female/male)	36/51	53/57	36/51	46/45
Age (mean ± SD)	14.93 ± 2.01	14.90 ± 2.07	14.93 ± 2.01	14.80 ± 2.10
BMI (mean ± SD)	21.71 ± 4.64	23.09 ± 5.40	21.71 ± 4.64	23.10 ± 5.66
Days post-injury (mean ± SD)	N/A	7.26 ± 2.28	N/A	130.08 ± 13.20
PCSI (mean ± SD)	5.86 ± 9.36	25.45 ± 25.94	5.83 ± 7.39	14.31 ± 19.48
PedsQL (mean ± SD)	N/A	N/A	87.12 ± 10.84	83.66 ± 13.04

SD represents standard deviation

**Fig. 2:**
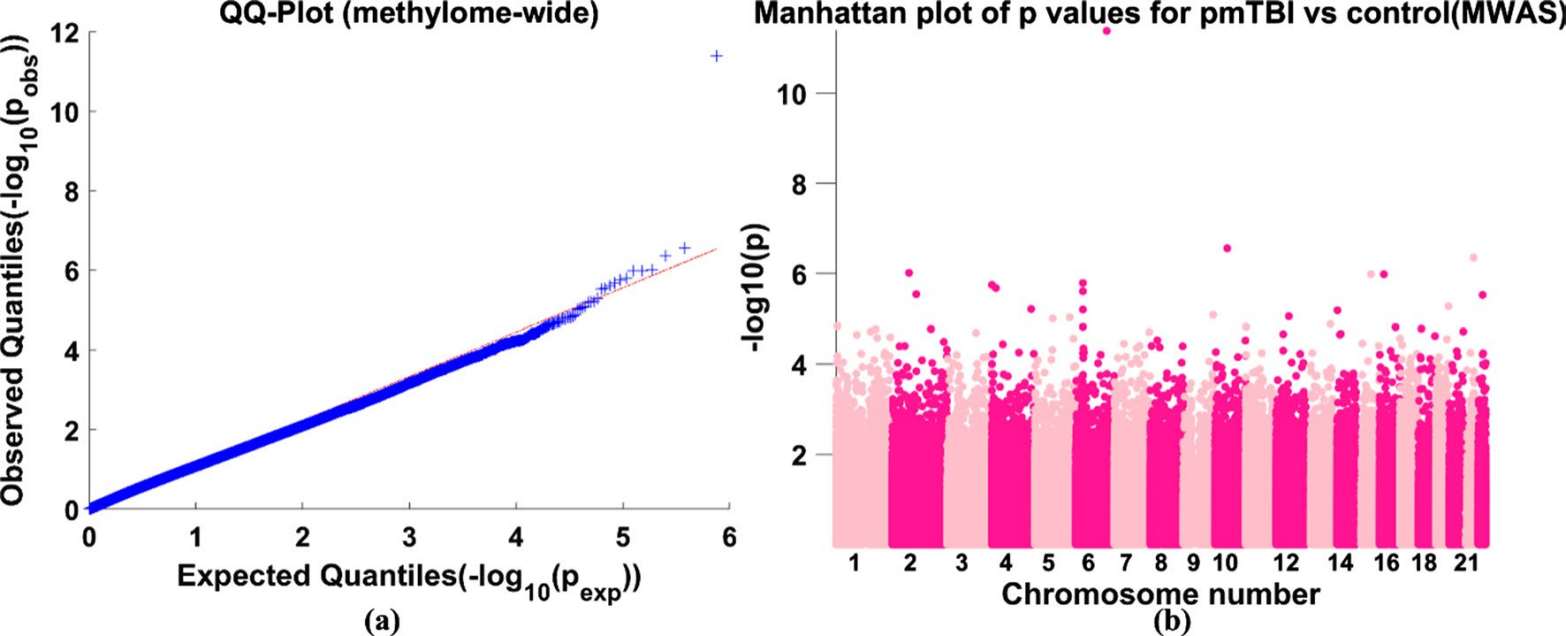
**a** Quantile–quantile plot of sorted − log10(*p*_obs_) values (ascending order, *p*_obs_ values were obtained from MWAS of 754,160 CpG sites for pmTBI versus control difference) against sorted − log10(*p*_exp_) values (ascending order, *p*_exp_ were sampled from a uniform distribution), **b** Manhattan plot of − log10 transformed *p* values for pmTBI versus control difference of 754,160 CpG sites

Figure 2a shows the quantile–quantile plot of the sorted MWAS *p* values for pmTBI vs control difference of 754,160 CpG sites against sorted *p* values sampled from a uniform distribution. The sorted MWAS *p* values largely followed a uniform distribution, indicating that there is no systematic bias. Figure 2b demonstrates the Manhattan plot of the MWAS *p* values for pmTBI versus control difference. One CpG site, cg00415333 located in chromosome 6 and in the open sea region of the protein-coding gene IL22RA2, showed significant hypermethylation in pmTBI patients compared to controls (*p* = 4.12 × 10^–12^, *t* = 7.42, degree of freedom = 186) after FDR at *p* < 0.05 correction for 754,160 CpG sites. Global methylation did not show a significant difference between pmTBI and HC groups.

### PCSI prediction

**Fig. 3:**
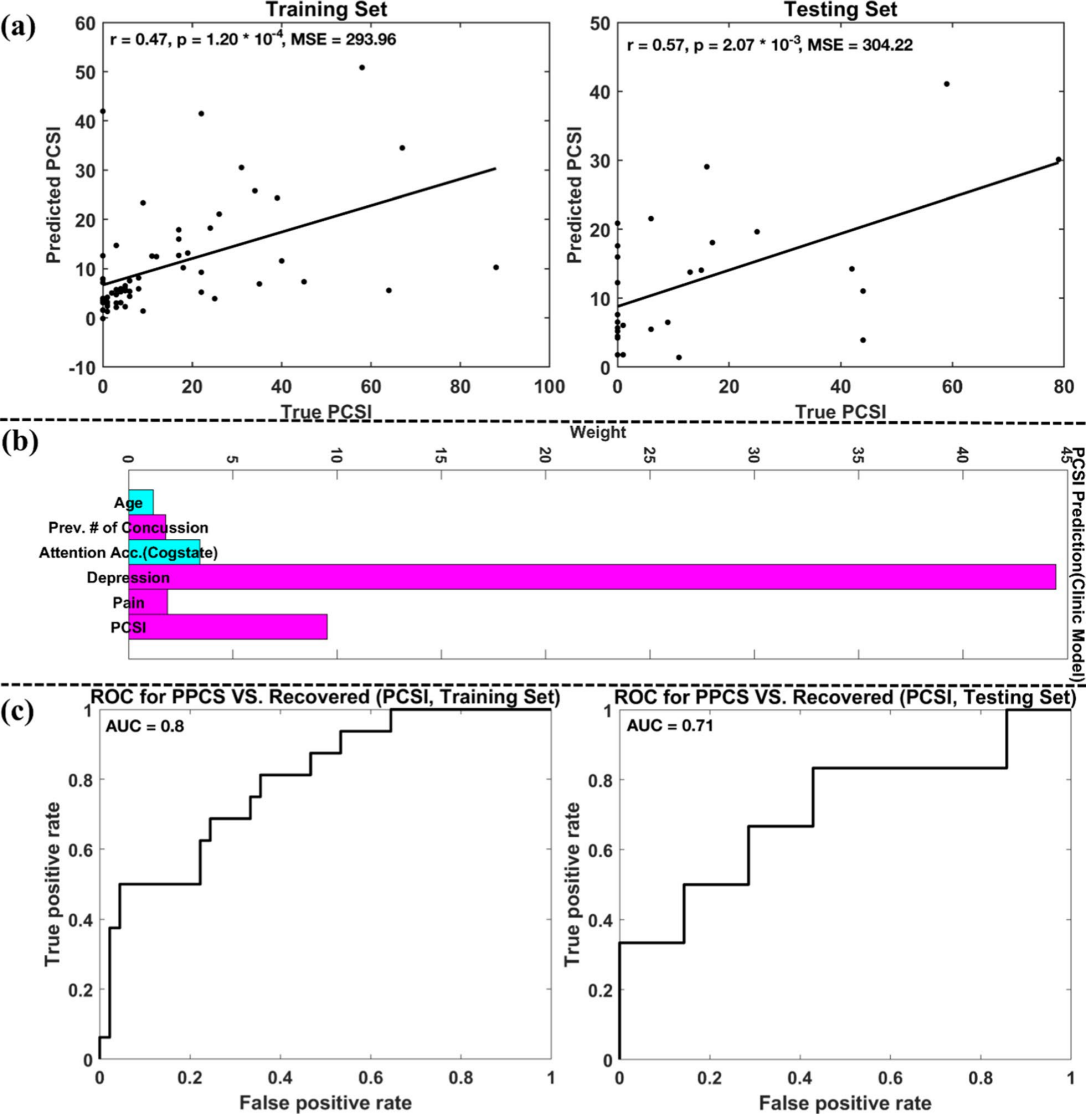
**a** Accuracy (correlation and MSE) and **b** weights of included variables for predicting PCSI using Model 1, **c** ROC curves and AUC values of classifying PPCS versus recovered patients using the predicted PCSI score from Model 1. Note, cyan and magenta colors denote negative and positive weights, respectively. Prev. # of Concussion represents the previous number of concussions, and Attention Acc. (Cogstate) denotes attention accuracy from the Cogstate test (the same for Fig. 4b)

Prediction accuracies of PCSI scores at EC are summarized in Table 3 upper panel. Model 1 (clinical model) selected six variables, including age, the previous number of concussions, attention accuracy from the Cogstate battery, depression score, pain scale, and the SA PCSI score. Model 1-predicted PCSI score was significantly and positively related to the true PCSI score for both training and testing sets (Fig. 3a). Using Model 2 (methylation model), the predicted PCSI was not significantly related to the true PCSI at EC (*p* > 0.5). Thus, Model 3 (clinical + methylation model) was the same as Model 1. Using Model 4 (prior clinical model) to predict PCSI at EC, the resulting PCSI score was significantly and positively related to the true PCSI score for the training set, but not for the testing, indicating a high likelihood of model overfitting. Using Model 5 (prior methylation model), the predicted PCSI was not significantly related to the true PCSI at EC visit for both training and testing sets (*p* > 0.4). The predicted PCSI from the methylation model, prior clinical and prior methylation models was not further tested for classification of PPCS.

**Table 3:** Accuracies for predicting PCSI/PedsQL using Models 1–5 and AUC values

Prediction	Models	Correlation		MSE		AUC for classifying PPCS versus recovered	
		Train	Test	Train	Test	Train	Test
PCSI prediction	Model 1	$r$=0.47, *p* = 1.20 × 10^–4^	$r$= 0.57, *p* = 2.07 × 10^–3^	293.96	304.22	0.8	0.71
	Model 2	N. S	N. S	N/A	N/A	N/A	N/A
	Model 4	$r$= 0.41, p = 8.00 × 10^–4^	N. S	340.28	458.34	N/A	N/A
	Model 5	N. S	N. S	N/A	N/A	N/A	N/A
PedsQL prediction	Model 1	$r$= 0.71, *p* = 1.76 × 10^–10^	$r$= 0.59, *p* = 1.20 × 10^–3^	120.78	132.67	0.70	0.56
	Model 2	$r$= 0.42, *p* = 6.53 × 10^–4^	$r$= 0.50, *p* = 6.80 × 10^–3^	137.15	161.60	0.59	0.68
	Model 3	$r$= 0.74, *p* = 1.72 × 10^–11^	$r$= 0.71, *p* = 3.89 × 10^–5^	106.24	106.75	0.70	0.63
	Model 4	$r$= 0.36, *p* = 3.90 × 10^–3^	N. S	167.96	194.41	N/A	N/A
	Model 5	N. S	N. S	N/A	N/A	N/A	N/A

N. S. denotes not significant. Accuracies were reflected by the correlation and MSE value between predicted PCSI/PedsQL and true PCSI/PedsQL values. AUC values were for classifying PPCS versus recovered using the predicted PCSI/PedsQL values

**Fig. 4:**
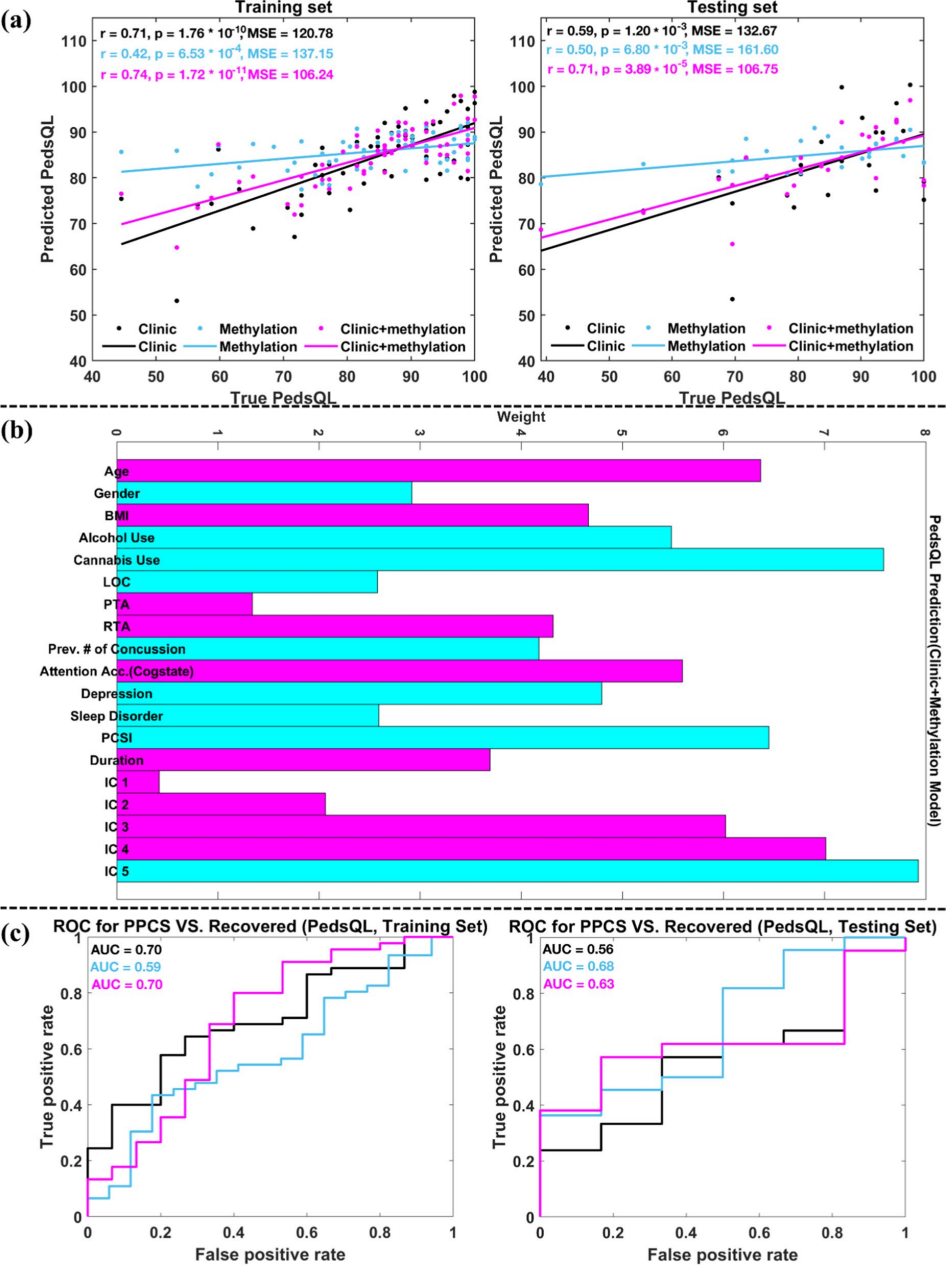
**a** PedsQL prediction accuracies (correlation and MSE) from Models 1–3, **b** weights of included variables for predicting PedsQL using Model 3, **c** ROC curves and AUC values of classifying PPCS versus recovered patients using the predicted PedsQL scores from Models 1–3 (black, light blue, and magenta lines represented the results for Models 1, 2, and 3, respectively)

Figure 3b plots the weights of six predictors identified in Model 1, where bars with cyan and magenta colors denote variables with negative and positive weights (same for Fig. 4b), respectively. Thus, individuals with younger age or lower attention accuracy at SA reported an increased symptom burden four-month post-injury. Participants with a history of previous concussion, or higher depression, pain scores, and symptom load at SA also reported an increased symptom burden four-month post-injury. Among the six selected variables, depression score had the largest weight, followed by PCSI at SA visit and attention accuracy from the Cogstate battery. Figure 3c displays the ROC curves of classifying PPCS versus recovered patients using the predicted PCSI score from Model 1 with all possible thresholds for training and testing sets, respectively. The AUC values under the ROC curves were 0.8 and 0.71 for training and testing data, respectively.

### PedsQL prediction

Results regarding the prediction of the PedsQL score at EC are summarized in Table 3 bottom panel. Model 1 (clinical model) selected fifteen variables including age, sex, BMI, the previous number of concussions, categories of LOC, PTA and RTA, depression score, sleep disorder, PCSI score at SA, attention accuracy from the Cogstate battery, the duration between two visits, as well as previous substance use of tobacco, alcohol, and cannabis. Based on these fifteen variables, the predicted PedsQL scores showed significant and positive relationships with the true PedsQL scores for both training and testing (Fig. 4a, black line). Model 2 (methylation model) selected loadings of 5 methylation components (ICs 1–5), and the predicted PedsQL scores (Fig. 4a, light blue line) demonstrated significant and positive associations with true PedsQL scores for both training and testing. Loadings of methylation ICs 1–5 and the fifteen variables selected from Model 1 were treated as final predictors in Model 3 (clinical + methylation model). The accuracy of the predicted PedsQL score was largely improved in Model 3 compared to Models 1 and 2 (see Fig. 4a, magenta line). Model 4 (prior clinical model) significantly predicted PedsQL at EC for the training set, but not for the testing set. Using Model 5 (prior methylation model), the predicted PedsQL was not significantly related to the true PedsQL at EC visit for both training and testing sets (*p* > 0.6). Thus, the results were not plotted, and the predicted PedsQL scores from the prior clinical and prior methylation models were not further tested for classification of PPCS.

The weights of the nineteen variables (tobacco use had a weight of zero, thus omitted) included in Model 3 (clinical + methylation model) for predicting PedsQL at EC are plotted in Fig. 4b. Age, BMI, categories of PTA and RTA, attention accuracy, duration between two visits, as well as loadings of methylation ICs 1–4 had positive weights, indicating that participants with larger values for these ten variables would have better outcomes four-month post-injury. However, sex (females were coded as ones, males were coded as zeroes), alcohol use, cannabis use, category of LOC, the previous number of concussions, depression score, sleep disorder, PCSI score at EA, and the loadings of methylation IC 5 had negative weights, implying that individuals with larger values for these nine variables would have worse outcome four-month post-injury. Among the nineteen variables, loadings of methylation IC 5 had the highest weight, followed by cannabis use, loadings of methylation IC 4, PCSI at EA, age, loadings of methylation IC 3, attention accuracy, alcohol use, and depression score, etc.

Figure 4c plots the ROC curves of classifying PPCS versus recovered patients using the predicted PedsQL scores from Models 1–3 with all possible thresholds for training and testing sets, where black, light blue, and magenta lines were used for Models 1 (clinical model), 2 (methylation model), and 3 (clinical + methylation model), respectively. The AUC values under training data ROC curves of Models 1–3 were 0.70, 0.59, and 0.70, respectively. The AUC values under testing data ROC curves of Models 1–3 were 0.56, 0.68, and 0.63, respectively. Using 91 pmTBI patients’ true PedsQL score to classify PPCS versus recovered patients, the AUC value was 0.74.

From Fig. 4a and c, we can observe that including methylation features decomposed from the major depression pathway largely increased the accuracy (reflected by the correlation coefficients and MSE values) of predicting PedsQL at EC for both training and testing data, and the predicted PedsQL scores yielded slightly higher accuracy of classifying PPCS versus recovered ones than that achieved from the clinical model for the testing set.

### Secondary analyses

Six univariate analyses were performed to test the association between each of the six variables selected from Model 1 (PCSI prediction) and the PCSI score at EC. No significant association was observed for any of the six variables. We also examined the association between the PedsQL score at EC and each of the nineteen predictors selected from Model 3 (PedsQL prediction). The result showed that only the depression score was significantly and negatively related to the PedsQL score at EC (*r* = − 0.48, *p* = 6.77 × 10^–5^) after FDR *p* < 0.05 correction, indicating that higher depression symptoms were associated with lower quality of life.

Among the six cognitive/clinical variables (not including LOC, PTA, RTA, PCSI, and pain scale due to obvious differences between controls and pmTBI) selected from Model 1 for PedsQL/PCSI prediction, tobacco and cannabis use, depression score and sleep disorder score showed significant pmTBI versus HC difference (see Table S3 for details). The results showed that pmTBI patients had higher usage of tobacco and cannabis, and higher depression and sleep disorder scores compared to HC. No significant group differences were observed for any of the five methylation components identified from Model 2 for PedsQL prediction or any of 13 CpG sites in BDNF and APOE4 genes.

Among the identified five methylation components, loadings of the pmTBI patients for methylation IC 3 significantly and positively related to PedsQL score at EC visit (*p* = 2.32 × 10^–3^, *R*^2^ = 6.92%), but negatively associated with PCSI score at EC visit (*p* = 3.75 × 10^–4^, *R*^2^ = 2.35%). Loadings of the pmTBI patients for methylation IC 5 significantly and negatively associated with the PedsQL score at EC (*p* = 4.80 × 10^–3^, *R*^2^ = 7.08%), but positively related to the PCSI score at EC (*p* = 2.39 × 10^–7^, *R*^2^ = 6.56%). No significant associations were observed between PCSI/PedsQL scores at EC visit and loadings of methylation ICs 1/2/4 or any of the 13 CpG sites in BDNF and APOE4 genes.

Within 91 pmTBI patients, neither the identified five methylation components nor the 13 CpG sites in BDNF and APOE4 genes were significantly related to the depression symptom score at EC visit. The identified five methylation components together significantly predicted the depression symptom score at SA (correlation between the predicted and true depression scores: *r* = 0.23, *p* = 1.68 × 10^–2^) and EC (*r* = 0.28, *p* = 6.90 × 10^–3^) visits for pmTBI patients. (Similar as regression model (d) in Table 1, the depression score at SA/EC visit was used as the dependent variable, with the loadings of the identified five methylation components, age, sex, BMI, and race used as predictors.) When using all 30 components from the major depression pathway to predict depression scores at SA and EC visit, the predicted score explained 16.81% of the variance within the true depression scores at SA visit (*r* = 0.41, *p* = 1.06 × 10^–5^, *R*^2^ = 16.81%), and 14.44% of the variance in the true depression scores at EC visit (*r* = 0.38, *p* = 2.26 × 10^–4^, *R*^2^ = 14.44%). Highlighted CpG sites (with weights |*z*|> 2) of identified methylation ICs 1–5, their locations and z values, relations to the island and annotated gene names are listed in Table 4.

**Table 4:** Annotations of highlighted CpG sites (|*z*|> 2) of methylation ICs 1–5

IC	CpG sites	Chr	BP Pos	*Z* value	Relation to Island	RefGene name (UCSC)
IC 1	cg19097407	chr9	36154750	14.42	OpenSea	GLIPR2
IC 2	cg19519355	chr8	26697488	12.53	OpenSea	ADRA1A
	cg10492858	chr21	35884679	2.43	OpenSea	KCNE1
	cg06279296	chr10	601816	− 2.48	OpenSea	DIP2C
	cg21163960	chr11	35441777	− 2.12	Island	SLC1A2
	cg22186155	chr8	72765158	− 2.02	OpenSea	MSC-AS1
IC 3	cg08549495	chr16	8823986	12.55	OpenSea	ABAT
	cg00549601	chr12	52208872	4.42	Island	
IC 4	cg03944460	chr4	3765186	12.82	N_Shore	
	cg27297376	chr7	98627840	2.70	OpenSea	SMURF1
	cg06279296	chr10	601816	− 2.64	OpenSea	DIP2C
IC 5	cg04306507	chr14	55594613	12.62	N_Shore	LGALS3
	cg09422301	chr6	24494043	− 3.37	N_Shore	ALDH5A1

‘Chr’ and ‘BP Pos’ represent chromosome number and base pair position, respectively. ‘RefGene Name (UCSC)’ denotes reference gene name from University of California, Santa Cruz (UCSC) genome browser

The top 12 unique CpG sites highlighted in ICs 1–5 (Table 4) were matched in the IMAGE-CpG database [50]. Five of these showed a significant correlation between brain methylation level and saliva methylation level (see details in Table 5). Based on the reliability summary in [51], four out of 12 top CpG sites were matched. cg06279296 (IC 2 and IC 4), cg00549601 (IC 3), and cg04306507 (IC 5) had excellent reliability (reliability > 0.8), and cg21163960 (IC 2) had bad reliability (reliability < 0.4).

**Table 5:** Correlation between saliva methylation level and brain methylation level for the top 5 CpG sites

IC	CpG sites	*r*	*p*
IC 1	cg19097407	0.61	3.88 × 10^–3^
IC 2	cg21163960	0.51	1.87 × 10^–2^
IC 3	cg08549495	0.44	4.93 × 10^–2^
	cg00549601	0.55	1.04 × 10^–2^
IC 5	cg04306507	0.63	2.55 × 10^–3^

*r* is the correlation value and *p* denotes the significance of the correlation

The MWAS on the depression score showed that no CpG sites were significantly associated with the depression score at SA after FDR at *p* < 0.05 correction. Using depressed-related top 34 CpGs (*p* < 0.01), no models trained could predict PCSI or PedsQL score at EC in the independent test data. See details in the Supplemental Materials.

Performing MWAS on PCSI score at EC, we obtained 14 CpG sites significantly related to PCSI score at EC in 63 training samples (FDR corrected for 17,857 CpG sites at *p* < 0.05). Using these 14 CpG sites to predict PCSI score based on SVR with LASSO, we achieved significant prediction on the training set (the predicated score was associated with the observed PCSI score with *r* = 0.52, *p* = 4.36 × 10^–5^), but not on the testing set (*p* = 0.49). MWAS on the PedsQL score detected no CpG sites significantly associated with PedsQL score at EC after FDR correction. Using PedsQL-related top 35 CpGs (*p* < 0.001), the predicted PedsQL score was significantly related to the observed PedsQL score on the training set (*r* = 0.84, *p* = 1.41 × 10^–17^), but not on the testing set (*p* = 0.61). See details in the Supplemental Materials.

## Discussion

This study investigated whether DNA methylation markers from major depression pathway could improve the prediction accuracies of outcomes following mTBI, precisely the post-concussive symptoms and quality of life four-month post-mTBI, in a pediatric cohort. We compared five different prediction models (Models 1–5: clinic, methylation, clinical + methylation, prior clinic, and prior methylation) using DNA methylation, and/or demographic and clinical measures. Our results demonstrated that DNA methylation factors under the major depression pathway contributed to the prediction of the quality of life but not persistent post-concussive symptoms. The prediction accuracy for quality of life, when including DNA methylation factors, was improved markedly compared to that obtained from only demographic and clinical measures. In contrast, global methylation did not improve the prediction performance, and did not show significant pmTBI versus control differences. Similarly, CpG sites in the most studied TBI-related genes, BDNF and APOE4, failed to predict both post-concussive symptoms and quality of life four-month post-injury.

When predicting post-concussion symptoms, the clinical model (using age, attention accuracy, previous concussion, depression, pain, and symptom load at SA) presented the best performance (correlation between observed and predicted values *r* = 0.57), with a prominent contribution from depression score. When predicting quality of life four-month post-injury, the clinical + methylation model demonstrated the highest accuracy (*r* = 0.71). The predictors include the same measures of the post-concussive symptom model above (except for pain) and additional clinical measures and five methylation factors. Not surprisingly, clinical symptoms are very important for predicting outcomes following TBI, as previously demonstrated in the Zemek model [21] and others [20]. Here, it is noteworthy that depression symptom score at SA contributed to prediction of both post-injury symptom and quality of life with relatively large weights (Fig. 3b and 4b). When adding DNA methylation factors from the major depression pathway, the prediction accuracy of quality of life was improved markedly from *r* = 0.59 (clinical model) to *r* = 0.71. One study led by Hellstrom on prediction of outcome after mTBI has reported a very similar prediction power (*r* = 0.55) from a clinical model also based on demographic and clinical measures, with no improvement in prediction found from additional MRI-based measures of brain morphometry [20]. In comparison, the added value we observed from DNA methylation indicates the strong relevance of both DNA methylation and depression.

The identified five methylation components under the major depression pathway highlighted 12 unique CpG sites and 10 unique genes. Five CpG sites, cg19097407 (IC 1), cg21163960 (IC 2), cg08549495 and cg00549601 (IC 3), and cg04306507 (IC 5) had high saliva-brain correspondence (i.e., had significant correlation between saliva methylation level and brain methylation level). Moreover, the methylation levels of cg06279296 (IC 2 and IC 4), cg00549601 (IC 3), and cg04306507 (IC 5) were very reliable (reliability > 0.8). The function of some identified genes has been related to TBI in several molecular studies. For instance, 4-aminobutyrate aminotransferase expression (ABAT), playing key roles in the biogenesis and metabolism of gamma-aminobutyric acid, was upregulated among other genes in adult zebrafish with mTBI 21-day post-injury [52]. Cellular expression of excitatory amino acid transporter 2 (also known as solute carrier family 1 member 2 (SLC1A2)) was reduced in humans after TBI, and the reduction is mainly caused by degeneration of astrocytes and downregulation in surviving astrocytes [53]. Expression of smad ubiquitination regulatory factor 1 (SMURF1) was low in the normal spinal cord and increased after acute spinal cord injury in adult rats. Expression of LGALS3 (galectin 3) was upregulated in adults’ plasma following mTBI [54] and in mice brain cortex and hippocampus after controlled cortical impact head injury [55]. Aldehyde Dehydrogenase 5 Family Member A1 (ALDH5A1, known as succinic semialdehyde dehydrogenase) is a mitochondrial homotetramer protein, and its expression showed a dynamic pattern from elevation at 1-day post-injury, followed by a reduction at 3- and 7-day post-injury and then restoration at 10-day post-injury in adult male rats [56]. Although the role of the alpha 1-adrenergic receptor (ADRA1A) in TBI is not consistent, its involvement is clear. Studies have reported that ADRA1A binding density was reduced in rats after experimental brain injury [57], and the reduction was progressed to the whole brain 30-day post-injury [58]. A more recent study revealed that increased ADRA1A mRNA expression level was related to working memory dysfunction in rats with TBI [59]. The MWAS highlighted cg00415333, which is annotated to the protein-coding gene IL22RA2. IL22RA2 is likely involved in the inflammatory response. The expression level of the IL22RA2 gene was shown to be downregulated in TBI plus vehicle-treated mice compared to sham plus vehicle-treated mice [60].

In the independent testing dataset, depression-related 34 top CpG sites (*p* < 0.01) failed to predict both PCSI and PedsQL scores at EC, the14 CpG sites significantly associated with PCSI failed to predict PCSI at EC, and PedsQL-related top 35 CpG sites (*p* < 0.001) failed to predict PedsQL at EC. Altogether these results suggest that prediction models trained with features selected from a relatively small sample MWAS results are not robust and generalizable. In contrast, depression-related methylation features selected from IPA, which have been verified by other studies, promise to be more generalizable in prediction models and can be easily implemented in other independent datasets.

Besides the molecular role of major depression in the recovery process following TBI, our results also highlighted that depression symptoms were a strong predictor for injury recovery following TBI. Specifically, higher depression symptoms were associated with greater post-concussive symptoms and lower quality of life. Previous studies have indicated that patients with major depression induced by TBI were reported to have significantly greater impairment in executive function and poorer social functioning 6- and 12-month post-injury [61], lower quality of life at one year [23], poorer performance on working memory, processing speed, verbal memory and executive function [62], higher degrees of psychological distress, psychosocial dysfunction, and post-concussive symptoms, and poorer instrumental activities of daily living performance compared to those without major depression following TBI [63].

Interestingly, DNA methylation factors under the major depression pathway demonstrated different powers in predicting post-concussion symptoms versus quality of life, while depression score was a strong predictor for both. We speculate that the reason may be twofold. One is that PCSI focuses on post-concussive symptoms, while PedsQL measures multidimensional functioning of pediatric life, including physical, emotional, social, and school. The other is that there are differences between DNA methylation under major depression pathway and depression scores. Altogether, 30 methylation factors in the major depression pathway only explained 16.81% of the variance within the depression score at SA visit in pmTBI patients. The five identified methylation factors together significantly predicted the depression symptom at both visits in pmTBI patients, but only explained 5% of the variance of the SA depression symptom and 8% of the variance of the EC depression symptom. Among the five identified methylation factors, two were associated with PedsQL score at EC in pmTBI individuals. These results indicate that DNA methylation factor from the major depression pathway partially underlies depression symptom presentation, as well as other domains of functioning. Depression scores assist prediction of both symptom and functional outcomes of mTBI, and specific methylation factors add additional prediction value for functional outcome.

Based on an empirical model [38], we classified patients with PPCS from “recovered” using self-reported PCSI scores at EC. We then compared the classification power for PPCS versus recovered using the predicted PCSI and PedsQL scores. The best prediction model (clinical model) of PCSI could classify PPCS from recovered with AUC of 0.71, and the best prediction model (clinical + methylation model) of PedsQL could classify PPCS from recovered with AUC of 0.63. Due to the lack of a standard definition for PPCS, these results cannot be directly compared to that in Zemek’s model [21]. However, the accuracy of classifying PPCS from recovered was comparable.

Global methylation changes following TBI have been reported in a few preclinical and human studies. One preclinical study reported that rats who experienced experimental TBI presented global hypomethylation in the brain in the early process following TBI (1–4-day post-injury) [64]. Another human epigenetic study focusing on a small population of college students (25 participants: 11 mTBI patients, and 14 controls) revealed that mTBI had a long-term effect on global methylation, with patients demonstrating a significantly higher blood global methylation compared to controls seven years post-injury [65]. Our lack of findings from global methylation could be from three factors. First, different populations may have different methylation change trajectories following mTBI. Zhang’s study focused on rats and Lee’s focused on college students seven years post-injury. We focused on pediatric participants, and global methylation change may not have yet happened when we collected the DNA samples at 7.26 ± 2.28-day post-injury. Second, different tissues may have different methylation change trajectories. Zhang’s study focused on rats’ brains, Lee’s focused on methylation profile from human blood, while we focused on methylation features from saliva which is more distant from the injury site. Third, different assays capture different aspects of global methylation. We used the Infinium Methylation EPIC array, while Zhang used immunohistochemistry and Lee employed the enzyme-linked immunosorbent assay. As such, under/over-estimation of global methylation may exist [66].

The current study should be considered in the context of strengths and limitations. This study leveraged raw demographic and clinical variables, as well as DNA methylation markers under the major depression pathway, which provided us more information to better estimate the post-concussion symptom and quality of life post-injury. The limitations may include that CPG sites identified from saliva methylation for predicting brain-related outcome (quality of life) may not be easily interpretable given that the underlying mechanism of how saliva relates to the brain is unknown or not direct. Some CpG sites have significant correlations between saliva methylation and brain methylation, such as the five important CpG sites highlighted in Table 5 (cg19097407, cg21163960, cg08549495, cg00549601, and cg04306507). Other CpG sites may predict the brain-related outcome through indirect and/or secondary pathways. Another limitation is that DNA analyzed in this study was collected from saliva, where the concentration and purity of DNA may be lower than that from the blood/brain. We performed a series of quality control to only include CpG sites with a higher (> 95%) detection probability and with variance (> 0.1) much larger than measurement errors. We may have excluded CpG sites with moderate variance (< 0.1) that could be relevant to PCSI and PedsQL predictions. It is also challenging to collect methylation data prior to the injury since we cannot predict the occurrence of injury. This limits our ability to definitively link the DNA methylation markers collected at the SA visit to depression symptoms introduced by the trauma or rule out the possibility that they existed prior to the injury. Importantly, these methylation markers are predictive of poor outcomes regardless of whether they occurred pre- or post-injury. A final limitation is the relatively modest sample size utilized in the analyses. Our findings should be interpreted with caution pending independent replication.

## Conclusions

In summary, the current study leveraged traditional demographic and clinical variables as well as DNA methylation markers under the major depression pathway to predict outcomes (i.e., post-concussive symptoms and quality of life) four months following mTBI in a cohort of 110 pmTBI patients and 87 age-matched healthy controls. The results highlighted that both molecular and behavioral manifestations of depression symptoms had a profound impact on the recovery trajectory following mTBI, suggesting the future direction of preventing TBI-caused symptoms for better recovery outcome.

## Supplementary Information

**Additional file 1.** Supplemental Materials.

**Additional file 2.** Genes and CpG sites included.

## Data Availability

The datasets analyzed in the current study are available from the corresponding author on reasonable request.

## Abbreviations

mTBI: Mild traumatic brain injury
CpG: Cytosine-phosphate-guanine
TBI: Traumatic brain injury
SNPs: Single-nucleotide polymorphisms
BDNF: Brain-derived neurotrophic factor
PPCS: Persistent post-concussion symptom
APOE: Apolipoprotein E
MDD: Major depressive disorder
pmTBI: Pediatric mild traumatic brain injury
HC: Healthy control
SA: Subacute
EC: Early chronic
PCSI: Post-Concussion Symptom Inventory
PedsQL: Pediatric Quality of Life Inventory
PROMIS: Patient Reported Outcomes Measurement Information System
HIT: Headache Impact Test
LOC: Loss of consciousness
PTA: Posttraumatic amnesia
RTA: Retrograde amnesia
ASSIST: Alcohol, Smoking, and Substance Involvement Screening Test
DKEFS: Delis–Kaplan Executive Function System
PC: Principal components
MWAS: Methylome-wide association study
SD: Standard deviation
BMI: Body mass index
IPA: Ingenuity pathway analysis
ICA: Independent component analysis
SVR: Support vector regression
LASSO: Least absolute shrinkage and selection operator
MSE: Mean square error
ROC: Receiver operating characteristic
AUC: Area under the curve
FDR: False discovery rate
Chr: Chromosome number
BP Pos,: Base pair position
ABAT: 4-Aminobutyrate aminotransferase
SLC1A2: Solute carrier family 1 member 2
SMURF1: Smad ubiquitination regulatory factor 1
ALDH5A1: Aldehyde Dehydrogenase 5 Family Member A1
ADRA1A: Alpha 1-adrenergic receptor
