# Supplementary material for: DNA methylation under the major depression pathway predicts pediatric quality of life four-month post-pediatric mild traumatic brain injury
Source: Clin Epigenetics. 2021 Jul 12;13:140. doi: 10.1186/s13148-021-01128-z (PMC8274037; doi:10.1186/s13148-021-01128-z)
Supplement: Supplementary file 1 — Additional file 1. Supplemental Materials. [file 13148_2021_1128_MOESM1_ESM.docx]

***Supplemental Materials***

*Participants*

One hundred and eleven pmTBI patients (8–18 years; 14.92±2.07 years old; 54 female) and 89 age- and sex-matched (14.97±2.00 years old; 38 female) healthy controls (HC) were selected from a larger, ongoing study examining the effects of pmTBI on imaging and fluid biomarkers (1). Inclusion criteria were based on the American Congress of Rehabilitation Medicine and the Zurich Concussion in Sport Group guidelines. Specifically, pmTBI were enrolled if they experienced a closed head injury with Glasgow Coma Score ≥ 13, loss of consciousness (if present) limited to 30 minutes, post-traumatic amnesia (limited to 24 hours), alteration in mental status at the time of injury, or at least two new post-concussion symptoms (PCS).

Exclusion criteria for all participants included a history of 1) previous head injury with greater than 30-minute loss of consciousness, 2) a neurological diagnosis, 3) history of any psychiatric disorders other than adjustment disorder, 4) developmental disorder (autism spectrum disorder or intellectual disability), 5) history of substance abuse/dependence, 6) contraindications for MRI including pregnancy, or 7) non-English speaking. Additional exclusion criteria for HC included a diagnosis of Attention-Deficit Hyperactivity Disorder, a learning disability, or a recent mTBI (within six months). Patients were also excluded if general anesthesia was administered immediately following injury or if the injury affected the dominant hand. Urine-based drug screens were conducted for all participants at each visit. Positive tests resulted in exclusion from the current analyses.

*Race Information of Included 197 Subjects*

Out of 197 subjects, 164 were White, 5 were Black or African American, 14 were American Indian or Alaska Native, 11 were mixed heritage, 2 were Native Hawaiian Or Other Pacific Islander, and 1 was Asian.

*DNA Extraction*

Participants were given approximately 15mL of mouthwash and instructed to swish vigorously for 30-60 seconds. Obtained saliva samples were then stored in a refrigerator for a maximum of 72 hours until they were processed. Samples were processed by centrifuging at 3400rpm at 4ºC for 30 minutes then removing supernatant. Pellets were washed in 10ml of cold PBS and centrifuged at 3400rpm at 4ºC for 5 minutes removing supernatant and repeating once. Pellets were stored at -80ºC until DNA was isolated. DNA was isolated by thawing the saliva pellets and samples were mixed with 515 μL of a solution containing 425μL TNE buffer, 25μL Proteinase K [10mg/mL], 50μL 10% SDS, 5μL RNAse A [10 mg/mL], and 10μL T1 RNAse [1250U/mL] before being rotated overnight at 55°C. Two sets of 2mL phase lock gel tubes were then centrifuged at max speed for one minute, with digested samples then flash. 500 μL of phenol was added, with the resulting solution placed in the centrifuge at max speed for an additional 5 minutes. The top aqueous layer was transferred to phase lock tubes, and then 250μL phenol and a 24:1 ratio chloroform/isoamyl alcohol solution were added. Tubes were then thoroughly mixed by hand to form a homogenous suspension followed by centrifuging at max speed for 5 minutes. The aqueous layer was again moved to the phase lock tubes and added to 500 μL of the 24:1 chloroform/isoamyl alcohol solution. The tubes were mixed until homogenous, and then again placed in the centrifuge for 5 minutes at max speed.

The resulting aqueous solution was transferred to new 1.7mL tubes, with 50μL 3M sodium acetate and 1mL 100% cold ethanol added. Samples were rotated to fully incorporate the solution and placed in a -80°C freezer for at least one hour. Samples were then spun at max speed for 30 minutes at 4°C. Supernatant was pulled off, the pellet was washed with 1mL of 70% cold ethanol, and then spun at max speed for 10 minutes. The ethanol was removed so the pellets could air dry, before finally being re-suspended in TE buffer. DNA was quantified by Qubit Assay and quality assurance metrics for DNA concentration levels included electrophoresis by running 500 nanograms of samples on a 1% agarose gel to confirm band.

*Clinical Data*

Categorical classification for LOC, PTA, retrograde amnesia, and confusion/disorientation were created based on self-report and were identical to a previous study in adults (2) using previously published (3, 4) categorical classifications. Specifically, self-report data for LOC category was classified in the following way: 0 = none, 1 = less than 1 minute, 2 = 1 minute to 5 minutes, 3 = 5+ minutes. 4 = 30+ minutes. The following classification was used for PTA and RTA self-report data: 0 = none, 1 = 0 to 5 minutes, 2 = 5 to 30 minutes, 3 = 30+ minutes, 4 = 24 hours or more.

The 5P clinical risk score was computed consistent with prior work from Zemek et al. (5), with two modifications. The typical calculation includes age group, sex, prior concussions and symptom duration, physician-diagnosed migraine history, answering questions slowly on the Acute Concussion Evaluation (ACE) physician/clinician office version, mBESS score in tandem stance, headaches on PCSI-Parent, sensitivity to noise on PCSI-Parent, and fatigue on PCSI-Parent. For each of the factors included, a severity score (0 to 2 points) was calculated, then pooled for getting a total risk score with a maximum of 12. In the current study, the mBESS score in tandem stance was not available for our sample; thus we used a proxy measure that was available, which is the tandem gait (1, 6). Further, because the ACE was not available for our sample, we used the item "answers questions more slowly" from the PCSI-P as a proxy measure.

*Secondary Analyses*

The loadings of controls ($\mathbf{A}_{c}$) were constructed with a projection method given the methylation data of controls ($\mathbf{X}_{c}$) and methylation sources ($\mathbf{S}_{p}$) decomposed from 110 pmTBI patients: $\mathbf{A}_{c}=\mathbf{X}_{c}S_{p}^{+}$, where $+$ is the pseudo-inverse operation.

Table S1, Retrospective rating of clinical information across groups (same 197 subjects as at the SA visit).

| Clinical variables | HC (87 subjects) | pmTBI (110 subjects) |
| --- | --- | --- |
| PCSI (Symptom, mean ± SD) | 8.34±11.54 | 15.65±20.66 |
| PedsQL (Outcome, mean ± SD) | 87.02±9.65 | 81.38±12.55 |

Note, SD represents standard deviation.

Table S2, CpG sites (standard deviation > 0.05) in genes BDNF and APOE 4.

| Gene | Chromosome | BP start | BP end | CpG |
| --- | --- | --- | --- | --- |
| BDNF | chr11 | 27657625 | 27657625 | cg12508693 |
|  | chr11 | 27661304 | 27661304 | cg04685076 |
|  | chr11 | 27694685 | 27694685 | cg07919246 |
|  | chr11 | 27695210 | 27695210 | cg15014679 |
|  | chr11 | 27696004 | 27696004 | cg18354203 |
|  | chr11 | 27701991 | 27701991 | cg18595174 |
|  | chr11 | 27716538 | 27716538 | cg25928860 |
|  | chr11 | 27718978 | 27718978 | cg20108357 |
|  | chr11 | 27721350 | 27721350 | cg06260077 |
|  | chr11 | 27723789 | 27723789 | cg02613510 |
|  | chr11 | 27741478 | 27741478 | cg22128379 |
|  | chr11 | 27742435 | 27742435 | cg10635145 |
| APOE 4 | chr19 | 45416910 | 45416910 | cg03793277 |

***Results***

Table S3, Group difference of identified 6 cognitive/clinical variables from model 1 for PCSI/PedsQL prediction.

| pmTBI vs HC difference (pmTBI=1, HC=0) | *p* | Beta |
| --- | --- | --- |
| Tobacco use | 1.21×10^-2^ | 6.78×10^-1^ |
| Alcohol use | 9.92×10^-1^ | 1.89×10^-3^ |
| Cannabis use | 2.63×10^-2^ | 9.44×10^-1^ |
| Attention accuracy | 6.86×10^-1^ | 7.07×10^-3^ |
| Depression score | 1.49×10^-3^ | 3.24 |
| Sleep disorder score | 9.48×10^-3^ | 2.91 |

*MWAS on depression score*

We performed a univariate MWAS on 197 samples included in this study by regressing each CpG site on the depression symptom at SA, including diagnosis, age, gender, BMI, race, buccal cell proportions, and PC 2-4 as fixed effects and family ID as a random effect. Figure S1 shows the Manhattan plot of -log10 transformed p values of associations between each CpG site and depression score at SA. No CpG sites showed a significant association with depression symptoms at SA after a false discovery rate (FDR) at *p* < 0.05 correction.


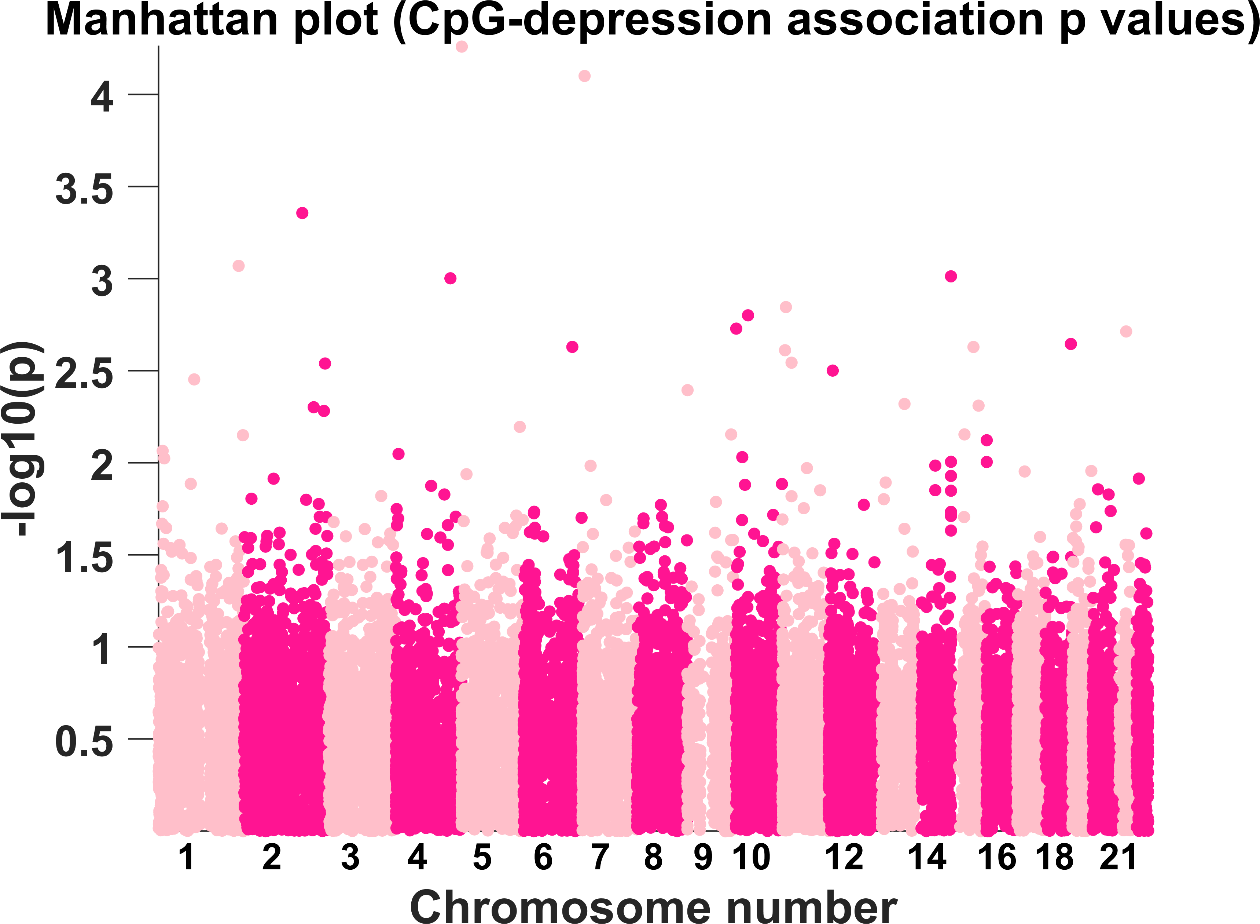


**Fig. S1.** The Manhattan plot of -log10 transformed p values of associations between each CpG site and depression score at SA.

*Predicting PCSI and PedsQL score at EC based on depression-related top CpG sites*

We used the top 34 CpG sites (*p* < 0.01) to predict the PCSI at EC by using SVR with LASSO (the same training and testing strategy as in the manuscript). The top 34 CpG sites didn’t include any CpG sites selected by IPA. The predicted PCSI score was significantly related to the observed PCSI score on the training set (correlation between predicted and observed PCSI scores: *r* = 0.35, *p* = 4.60×10^-3^, mean square error (MSE) = 399.56), but not on the testing set (*p* > 0.5). Similarly, we achieved the highest accuracy for PedsQL prediction on the training set (*r* = 0.53, *p* = 5.83×10^-6^, MSE = 124.95), but not on the testing set (*p* > 0.4).

*MWAS on PCSI score at EC and predicting PCSI score at EC*

Figure S2 displays the Manhattan plot of -log10 transformed p values of associations between each CpG site and PCSI at EC in 63 training samples. 14 CpG sites (Table S4) showed significant association with PCSI score after FDR at *p* < 0.05 correction.


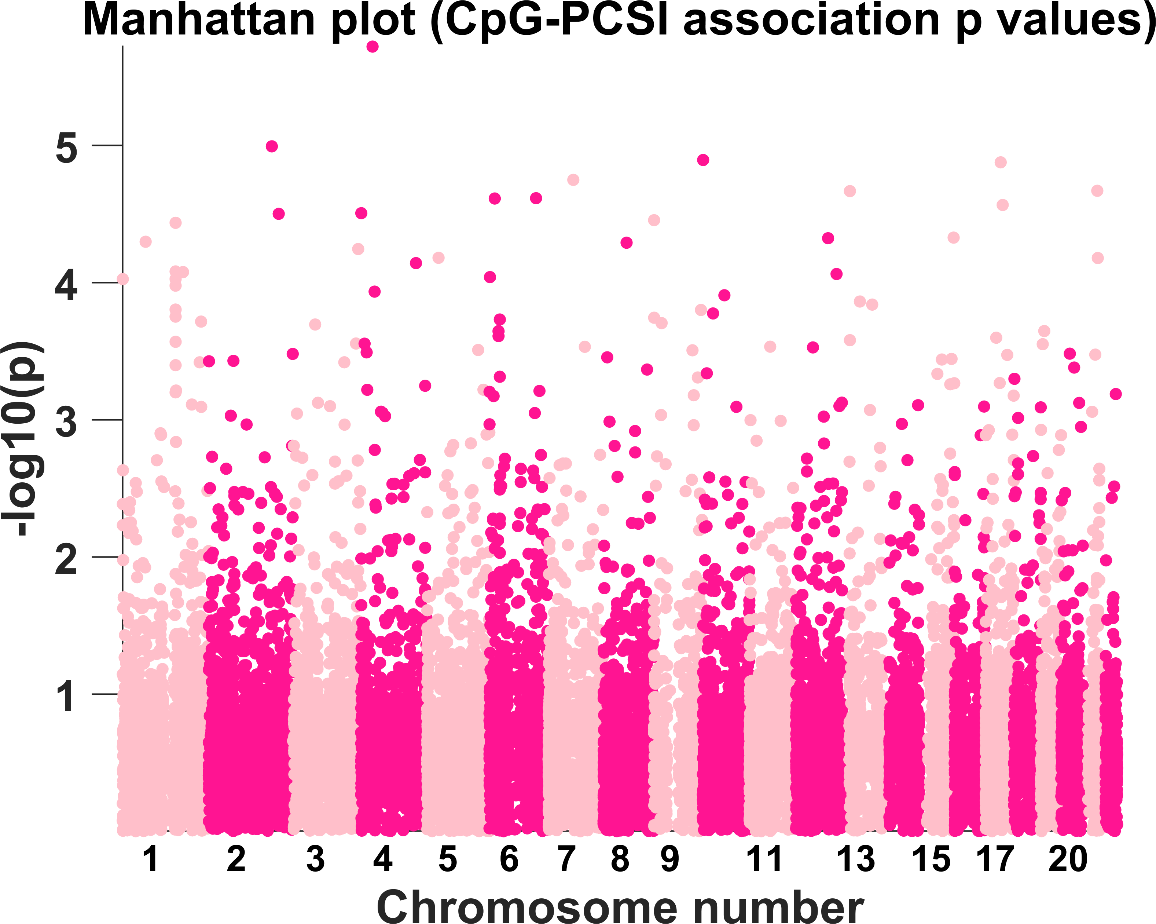


**Fig. S2.** The Manhattan plot of -log10 transformed p values of associations between each CpG site and PCSI score at EC in 63 training samples.

Table S4, 14 CpG sites significantly associated with PCSI at each in 63 training samples, their association p and t values.

| CpG-PCSI association | p | t |
| --- | --- | --- |
| cg02873163 | 3.66×10^-5^ | -4.52 |
| cg10660319 | 1.01×10^-5^ | -4.89 |
| cg22086510 | 3.15×10^-5^ | -4.56 |
| cg17035899 | 3.11×10^-5^ | -4.56 |
| cg20840591 | 1.90×10^-6^ | 5.36 |
| cg20849099 | 2.43×10^-5^ | 4.64 |
| cg02088676 | 2.42×10^-5^ | 4.64 |
| cg19757435 | 1.78×10^-5^ | -4.73 |
| cg13595143 | 3.50×10^-5^ | -4.53 |
| cg27336068 | 1.27×10^-5^ | -4.82 |
| cg00035636 | 2.15×10^-5^ | -4.67 |
| cg25538415 | 1.33×10^-5^ | 4.81 |
| cg20451208 | 2.71×10^-5^ | -4.60 |
| cg16731079 | 2.14×10^-5^ | 4.67 |

Note, the degree of freedom was 52 for all associations.

We used these 14 CpG sites to predict the PCSI score (same prediction strategies as in the manuscript except that the regularizer lambda was tuned with five-fold cross-evaluation). The predicted PCSI score was significantly related to the observed PCSI score on the training set (*r* = 0.52, *p* = 4.36×10^-5^, MSE = 323), but not on the testing set (*p* =0.49).

*MWAS on PedsQL score at EC and predicting PedsQL score at EC*

Figure S3 demonstrates the Manhattan plot of -log10 transformed p values of associations between each CpG site and PedsQL score at EC in 63 training samples. No CpG sites showed significant associations with PedsQL score at EC after FDR at *p* < 0.05 correction.


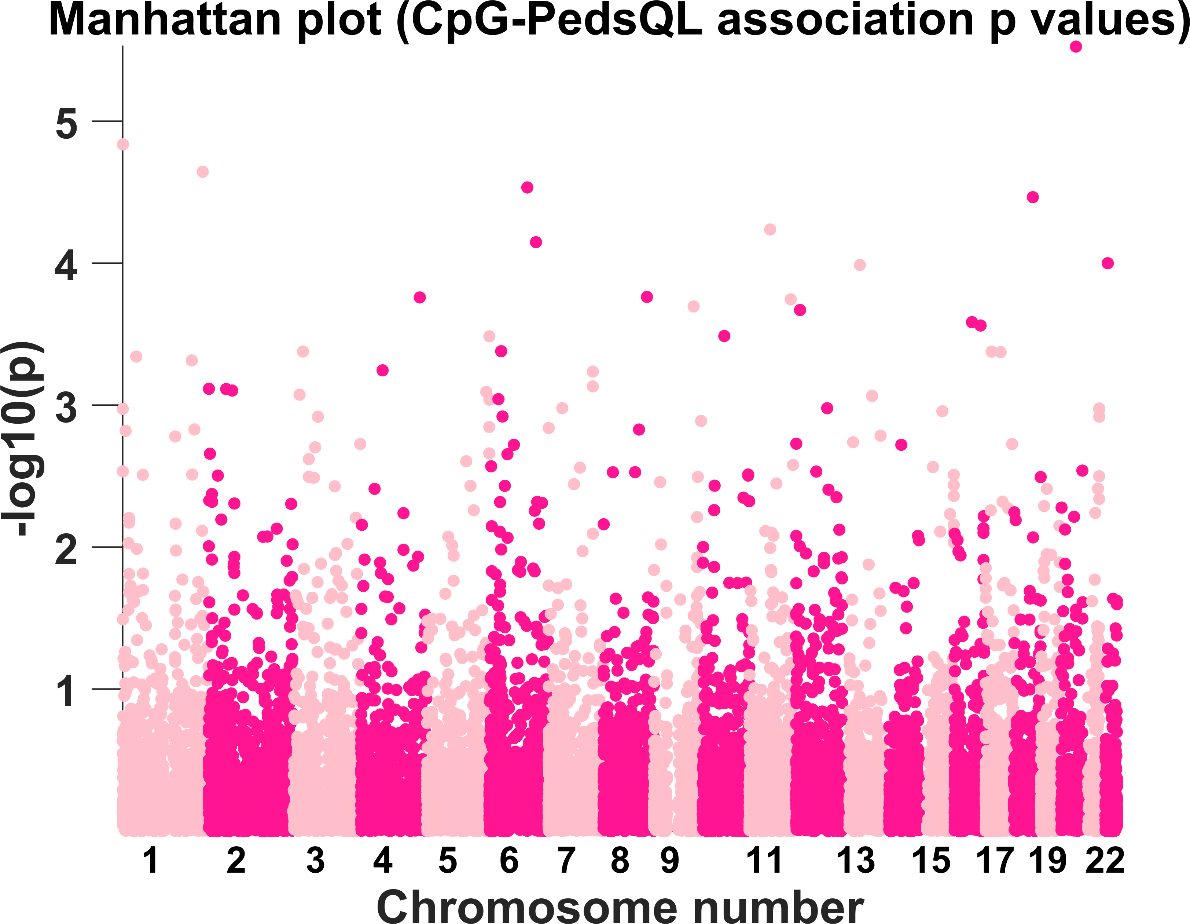


**Fig. S3.** The Manhattan plot of -log10 transformed p values of associations between each CpG site and PedsQL score at EC in 63 training samples.

The top 35 CpG sites (*p* < 0.001) were selected to predict the PedsQL score (same prediction strategy as in PCSI prediction mentioned above). The predicted PedsQL score was significantly related to the observed PedsQL score on the training set (*r* = 0.84, *p* = 1.41×10^-17^, MSE = 48.31), but not on the testing set (*p* = 0.61). Altogether, the results suggest that performing feature selection on 17,857 CpG sites with 63 training samples may lead to overfitting of the prediction model and yield low generalizability.

**Reference**

1. Mayer AR, Stephenson DD, Wertz CJ, Dodd AB, Shaff NA, Ling JM, et al. Proactive inhibition deficits with normal perfusion after pediatric mild traumatic brain injury. Hum Brain Mapp. 2019;40(18):5370-81.

2. Mayer AR, Hanlon FM, Claus ED, Dodd AB, Miller B, Mickey J, et al. An examination of behavioral and neuronal effects of comorbid traumatic brain injury and alcohol use. Biological Psychiatry: Cognitive Neuroscience and Neuroimaging. 2018;3(3):294-302.

3. Lezak MD. Neuropathology for neuropsychologists. Neuropsychological Assessment1995. p. 170-276.

4. Ruff RM, Jurica P. In search of a unified definition for mild traumatic brain injury. Brain Inj. 1999;13(12):943-52.

5. Zemek R, Barrowman N, Freedman SB, Gravel J, Gagnon I, McGahern C, et al. Clinical Risk Score for Persistent Postconcussion Symptoms Among Children With Acute Concussion in the ED. Jama-J Am Med Assoc. 2016;315(10):1014-25.

6. Mayer AR, Stephenson DD, Dodd AB, Robertson-Benta CR, Pabbathi Reddy S, Shaff NA, et al. Comparison of Methods for Classifying Persistent Post-Concussive Symptoms in Children. J Neurotrauma. 2020;37(13):1504-11.
